# A complete dietary review of Japanese birds with special focus on molluscs

**DOI:** 10.1038/s41597-021-00800-6

**Published:** 2021-01-20

**Authors:** Yuta Morii, Munehiro Kitazawa, Theodore E. Squires, Megumi Watanabe, Yoshiaki Watanabe, Takumi Saito, Daishi Yamazaki, Akitomo Uchida, Yoshiyasu Machida

**Affiliations:** 1 The Hakubi Project, Kyoto University, Oiwakecho, Kitashirakawa, Sakyo-ward, Kyoto, 6068501 Japan; 2 Laboratory of Animal Ecology, Department of Zoology, Graduate School of Science, Kyoto University, Oiwakecho, Kitashirakawa, Sakyo-ward, Kyoto, 6068502 Japan; 3 Graduate School of Agriculture, Hokkaido University, Nishi 9, Kita 9, Kita-ward, Sapporo, Hokkaido, 0608589 Japan; 4 Kenkyu Services, 12511 84th Ave NE, Kirkland, WA 98034 United States of America; 5 Komabakita 4-5-5, Abashiri, Hokkaido, 0930033 Japan; 6 Department of Biology, Faculty of Science, Toho University, Miyama 2-2-1, Funabashi, Chiba, 2748510 Japan; 7 Center for Northeast Asian Studies, Tohoku University, 41 Kawauchi, Aoba-ward, Sendai, Miyagi 9808576 Japan; 8 Tokiwacho 3-16-71, Kitami, Hokkaido, 0900817 Japan; 9 Bihoro Museum, Midori 253-4, Bihoro, Hokkaido, 0920002 Japan

**Keywords:** Food webs, Animal behaviour

## Abstract

Birds often hold important positions in the food webs of ecosystems. As a result, interactions between birds and their prey have attracted attention not only in ecology, but also in fields like agriculture and conservation. Avian food resources are well researched in Japan, however there is no database critically reviewing molluscs as a food resource for birds. Here, we present a new database reviewing dietary information for all Japanese bird species. In addition to addressing general diet categories and specific food habits for each bird, we include detailed data on the molluscan prey observed for all species that consume them. The information within this database was collected through intense literary review to provide a complete look at bird species historically present around the country. We also include new information on snail species found in the upper digestive tract of harvested wild birds. This database is publicly available in the Zenodo repository. The information should aid research around the Japanese archipelago, especially projects involving birds or molluscs.

## Abstract (summary)

**Table:** 

Measurement(s)	Distribution • endemicity • species status • habitat • diet • information of molluscan prey
Technology Type(s)	digital curation
Sample Characteristic - Organism	Aves • Mollusca
Sample Characteristic - Environment	sea • fresh water • terrestrial biome
Sample Characteristic - Location	Japan

Machine-accessible metadata file describing the reported data: 10.6084/m9.figshare.13296275

## Background & Summary

Ornithology has long been one of the main fields of naturalist study and significant knowledge has been accumulated during its long history^1,2^. Birds have some advantages for scientific research: (1) compared to other taxa, birds are easier to detect and identify via vocalization and are often less cryptic with fewer overall species than other macroorganisms^3^; (2) the occurrence, abundance, and reproductive success of birds has been shown to respond to environmental changes over many spatial scales^3^; (3) birds are often very popular flagship species for conservation and important for government policies^4,5^; and (4) the large volume of accumulated ornithological knowledge allows for strong review and cross-referencing of new research^6,7^.

Birds assume many fundamental ecological functions in the maintenance of local to continental-scale ecosystems. Their roles include dispersion of seeds, pollination, and long distance transfer and/or deposition of nutrients^8–12^. At the same time, birds are often in important positions as keystone and/or umbrella species in various community food webs^13–17^. In particular, the predator-prey interactions between birds and insects are well known; more than 50% of avian species are predominantly insectivorous, and nearly 75% prey on invertebrates at least occasionally^2,8,16^. Birds can reduce the densities of herbivorous insects in agriculture and may exert top-down effects on primary producers to the extent that their removal instigates trophic cascades^18,19^. Various investigations have shown that birds contribute significantly to agricultural success especially where they prey upon insects that are detrimental to human activities^17,20^. Thus, it is important to understand basic information about the food preferences of each bird species not only for ecological science but also for practical applications.

It is well established that birds prey on molluscs in various environments. Many species of sea ducks (Anatidae, tribe Mergini) prey on bivalves and have economic impacts on mussel farms worldwide^21^. The Snail Kite (*Rostrhamus sociabilis*; Accipitridae, Accipitriformes) and Limpkin’s (*Aramus guarauna*; Aramidae, Gruiformes) diet consists almost entirely of freshwater snails in the Americas^22–26^. Even for land snails, omnivorous birds are considered as regular predators (*e.g. Turdus* spp.; Muscicapidae, Passeriformes)^27–30^. Another example, the Great Tit (*Parus major*; Paridae, Passeriformes) and some other forest passerines in Eurasia take land snails as the main calcium source for eggshell production^28^. Despite these well documented instances, the scope of interactions between birds and molluscs are much less surveyed than between birds and insects.

Avian ecological databases have been established in several regions (*e.g*. Europe^31^, North America [https://www.audubon.org], Australia [https://birdata.birdlife.org.au], New Zealand [http://nzbirdsonline.org.nz], the North Pacific^32^, and Antarctica^33^) while others have global scope (*e.g*. BirdLife International [http://www.birdlife.org], Avibase [https://avibase.bsc-eoc.org], AS@S [http://seabirds.saeon.ac.za], and Birds of the World [https://birdsoftheworld.org]). Several platforms for citizen science also contribute to avian knowledge (*e.g*. eBird [https://ebird.org], iNaturalist [https://www.inaturalist.org] and GBIF [https://www.gbif.org]). However, most of these databases mainly focus attention on avian distributions, and often do not provide great detail on dietary preferences or predator-prey interactions^24,27^. Moreover, very few databases recorded prey species in these categories instead providing general overviews of diet.

Among regions there tends to be strong observation and research biases dependent on population, economy, language, and resource availability. These area-based biases can skew understandings of real diversity on earth and prevent comprehensive insights from being made. In this study, we investigated the diet of all bird species in Japan, with a special focus on molluscan prey. The Japanese archipelago and associated islands are located from the subarctic to the subtropical zone, therefore this area has high biodiversity and the region is recognized as a hotspot for the planet^34^. Similarly to other regions, ornithology in Japan has a long history and rich data to work from, but few researchers have reviewed the food preferences of all local bird species. Moreover, many publications reporting on the food composition of Japanese birds were written in Japanese, so it is difficult to disseminate this knowledge widely given the language barrier. There are some excellent works and databases showcasing the ecological traits of the entire avian fauna of Japan^35–38^, but they are only available in Japanese.

Most databases fail to identify molluscan prey at the species level despite wide variability within the phylum. It is important to determine the exact interactions between predator and prey at the species level due to ecological differences and unique conservation considerations for each party to a predatory interaction. In this study, we attempted to classify the main diet of all Japanese bird species, list detailed information on molluscs in avian diets, and evaluate molluscs as a food resource for birds based on available literature. We also newly record five species of land snails in the crop and gizzard of two species of birds hunted on Hokkaido, the northernmost island of Japan. These data are also included in our up-to-date database.

## Methods

### Classification of dietary preferences and habitats for bird species in Japan via literature

**Fig. 1:**
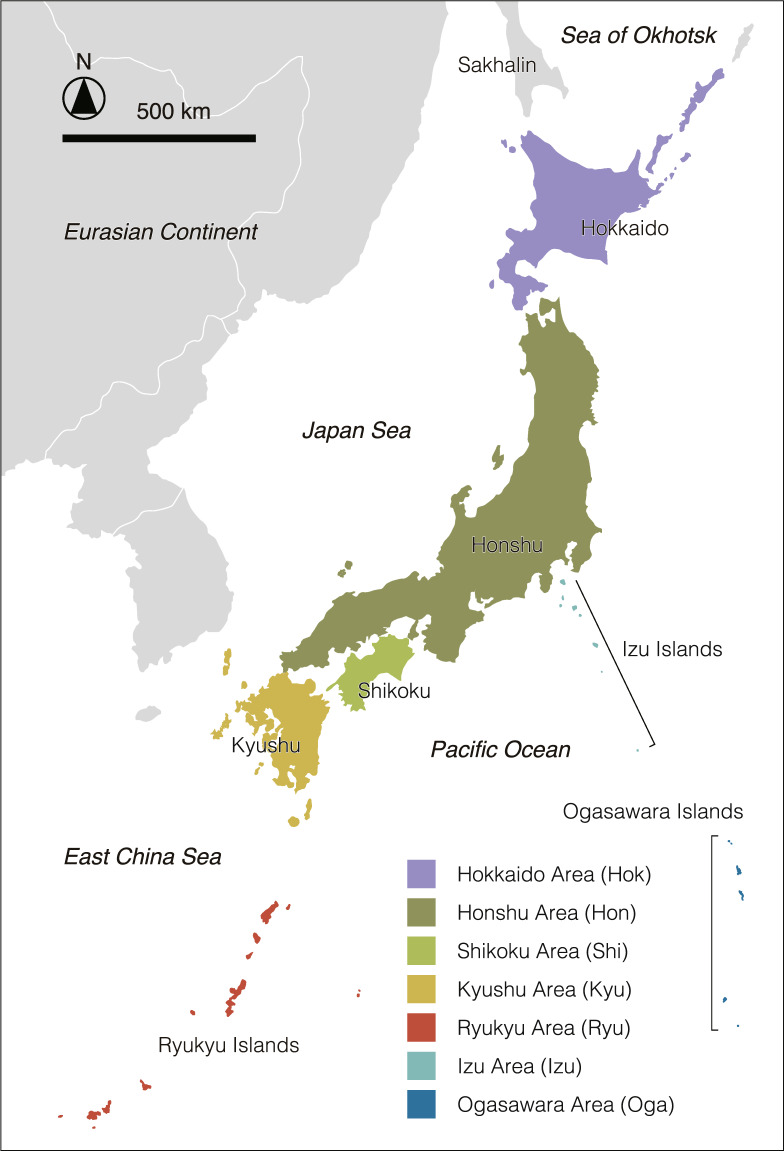
Seven categories of distribution area in this study.

**Fig. 2:**
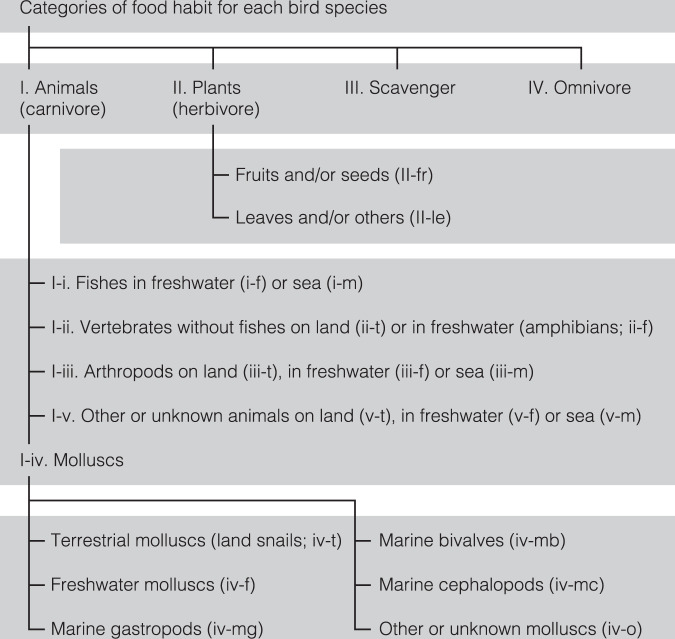
The categories of preferred foods in this study. Food preferences were first categorized into four big groups (I. carnivore, II. herbivore, III. scavenger and IV. omnivore), and two of them (I and II) were further separated. In particular, molluscs were classified in detail.

We reviewed the food habits of 633 native avian species listed in the Check-list of Japanese Birds, 7th Revised Edition^39^ in attempting to represent the whole avian fauna of Japan. Nine ecological traits related to distribution, habitat and diet are listed in our database along with references as shown below (Table 1): (1) the distribution and breeding status in each region of Japan (Fig. 1), (2) the endemicity in Japan (Endemic, or −: not endemic to Japan)^39^, (3) the species status in the Red List of Threatened Species of Japan, (4) main habitat (Terrestrial, Freshwater, and/or Marine, or Unknown), (5) dietary categories (I: carnivore, II: herbivore, IV: omnivore, or Unknown; Fig. 2), (6) main diet(s) (I: some animals, II: some plants, I-i: fishes, I-ii: vertebrates, I-iii: arthropods, I-iv: molluscs, I-v: unknown or other animals, II-fr: plants [fruits and/or seeds], and/or III: scavenger, or Unknown; Fig. 2), (7) all recorded food habits (I-i, I-ii, I-iii, I-iv, I-v, II-fr, II-le: plants [leaves and/or others], or III; Fig. 2), (8) molluscs as avian food resources (iv-t: terrestrial molluscs, iv-f: freshwater molluscs, iv-mg: marine gastropods, iv-mb: marine bivalves, iv-mc: marine cephalopods, or iv-o: others or unknown molluscs; Fig. 2), (9) descriptions of molluscan prey in literature, and (10) referenced bibliographies.

**Table 1:** Description of each variable, and factor levels.

Ten variables treated in the database	Explanations of factor labels for each variable
1. the distribution and breeding status	The bird distribution and residency in each area of Japan. **RB**: resident breeder, **MB**: migrant breeder, **WV**: winter visitor, **PV**: passage visitor, **FB**: former bred, or **−**: not distributed, rare or unknown (see also Fig. 1).
2. the endemicity in Japan	**Endemic**, or **−**: not endemic to Japan.
3. the species status in the Japanese Red List	The seven categories of species status based on the 2020, 4th Version of the Japanese Red Lists. **EX**: extinct, **CR**: critically endangered, **EN**: endangered, **VU**: vulnerable, **NT**: near threatened, **DD**: data deficient, or **−**: common species or not listed.
4. main habitat	Main habitat. **Terrestrial**, **Freshwater**, and/or **Marine**, or **Unknown**.
5. dietary categories	**I**: carnivore, **II**: herbivore, **IV**: omnivore, or **Unknown** (see also Fig. 2).
6. main diet(s)	Main diet(s). **I**: some of animals, **II**: some of plants, **I-i**: fishes, **I-ii**: vertebrates, **I-iii**: arthropods, **I-iv**: molluscs, **I-v**: unknown or other animals, **II-fr**: plants (fruits and/or seeds), **III**: scavenger, or **Unknown** (see also Fig. 2).
7. all recorded food habits	All recorded food habits of each bird species. **I-i**: fishes, **I-ii**: vertebrates, **I-iii**: arthropods, **I-iv**: molluscs, **I-v**: unknown or other animals, **II-fr**: plants (fruits and/or seeds), **II-le**: plants (leaves and/or others), or **III**: scavenger (see also Fig. 2).
8. molluscs as avian food resources	Six categories of molluscs as avian food resources (I-iv). **iv-t**: terrestrial molluscs, **iv-f**: freshwater molluscs, **iv-mg**: marine gastropods, **iv-mb**: marine bivalves, **iv-mc**: marine cephalopods, or **iv-o**: others or unknown molluscs (see also Fig. 2).
9. descriptions of molluscan prey in literature	The descriptions of molluscan prey and their taxonomies in literature.
10. referenced bibliographies	Referenced papers and books (shown in the reference list on Zenodo).

To keep our findings relevant, we reviewed the validity of species binomial names listed in our database and provide updates reflective of current taxonomic knowledge in 2020. A review was conducted using the Birds of the World online research database^36^ and apparent updates to binomials were cross-referenced using the International Union for Conservation of Nature’s Red List of Threatened Species (https://www.iucnredlist.org). The updated binomial information is included in Online-only Table 1.

We roughly categorised seven regions for avian distribution in the Japanese archipelago (Hok: Hokkaido Island and/or surrounding islands, Hon: Honshu Island and/or surrounding islands, Shi: Shikoku Island and/or surrounding islands, Kyu: Kyushu Island and/or surrounding islands, Ryu: Ryukyu archipelago, Izu: Izu islands, Oga: Ogasawara islands; Fig. 1), and classified six categories for residency in each region of Japan (RB: resident breeder, MB: migrant breeder, WV: winter visitor, PV: passage visitor, FB: former breeder, or −: not distributed, rare, or unknown) based on the Check-list of Japanese Birds, 7th Revised Edition^39^, and added seven categories for the species status in Japan based on the 2020, 4th Version of the Japanese Red Lists (EX: extinct, CR: critically endangered, EN: endangered, VU: vulnerable, NT: near threatened, DD: data deficient, or −: common species or not listed)^5^. To determine each species’ main diet, we primarily focused on literature describing “preferred” or “main” food habits, although we also utilized information about the frequency of target foods in crop and gizzard contents. The taxonomies of molluscan prey written in the database were mainly based on MolluscaBase (http://www.molluscabase.org), the online database of world mollusc classifications. While our database does not contain perfect information on distribution, residency, and conservation status in terms of current knowledge, we believe it represents a high degree of accuracy and usefulness in pulling together comprehensive information from different sources.

The diet data in this study was collected from 165 scientific articles and books including dietary information on Japanese birds. We searched for the following two series of keywords in Google Scholar for each bird species: {“scientific name” AND [“food habits” OR “diet” OR “food habits (in Japanese)” OR “crop and gizzard contents (in Japanese)”]} and {“standard Japanese name (in Japanese)” AND [“food habits” OR “diet” OR “food habits (in Japanese)” OR “crop and gizzard contents (in Japanese)”]}. Keyword searching and browsing was conducted between 2nd May and 27th December in 2017, and the top one-hundred and all results for each series of keywords was checked, respectively. Moreover, we manually reviewed additional several literatures and books as possible. These included publications in English and Japanese and were published between 1913 and 2018. All 165 references citing the food habits for each bird species are recorded in the database, and listed on the reference list in Zenodo^40^.

### Land snails detected from the crop and gizzard of two bird species in Hokkaido, Japan

**Fig. 3:**
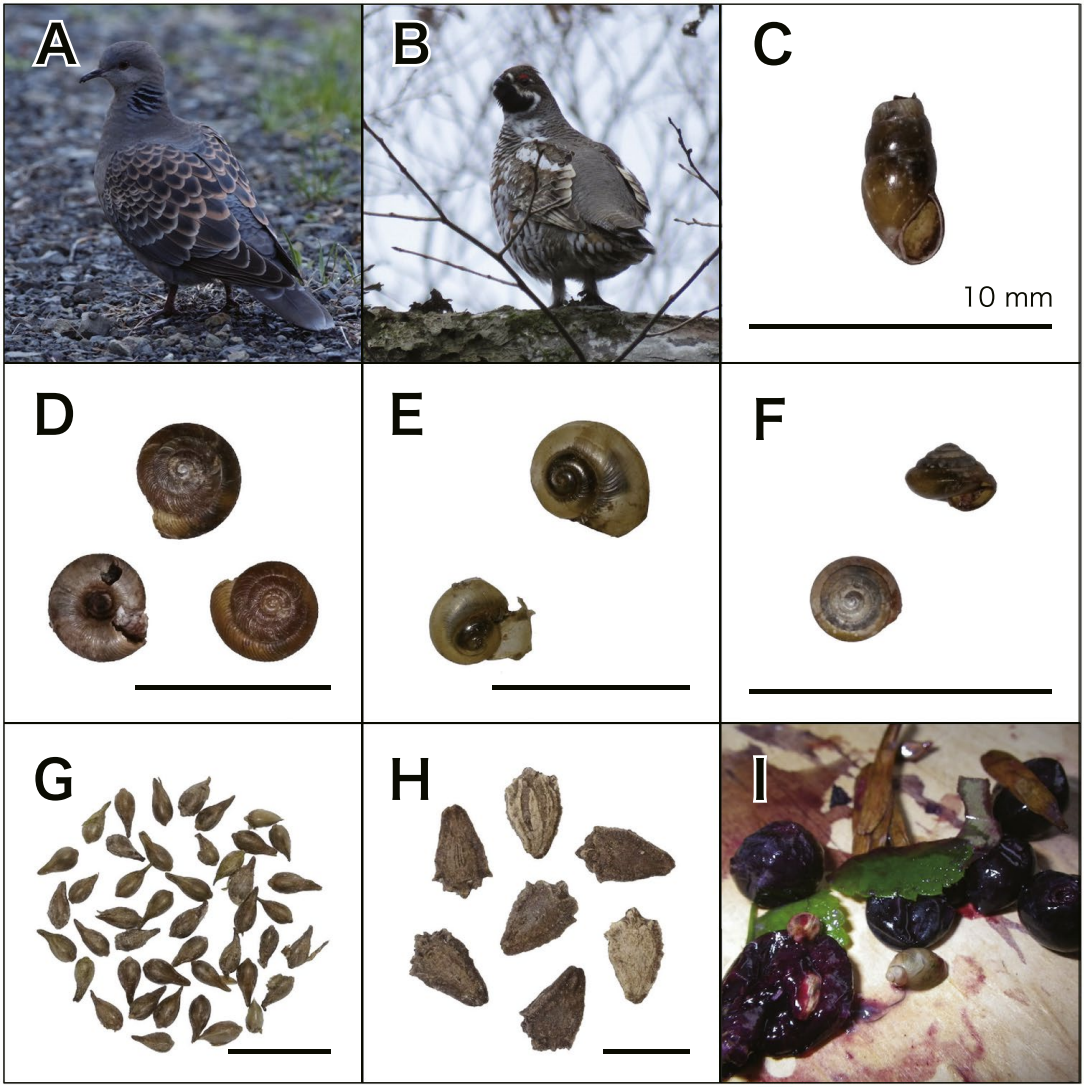
(**A**,**B**) Two bird species investigated in this study, *Streptopelia orientalis* (**A**), and *Tetrastes bonasia* (**B**). (**C**–**H**) The prey items detected from avian crops and gizzards of *S. orientalis*, (**C**) *Cochlicopa lubrica* (Cochlicopidae, Stylommatophora), (**D**) *Discus pauper* (Discidae, Stylommatophora), (**E**) *Karaftohelix* (*Ezohelix*) *gainesi* (Camaenidae, Stylommatophora), (**F**) *Parakaliella affinis* (Helicarionidae, Stylommatophora), (**G**) *Persicaria thunbergii* (Polygonaceae, Caryophyllales), and (**H**) *Schizopepon bryoniifolius* (Cucurbitaceae, Cucurbitales). I. The photograph of crop and gizzard contents of *T. bonasia*.

Crop and gizzard samples were obtained from two juvenile Oriental Turtle-Doves (*Streptopelia orientalis*; Columbidae, Columbiformes; Fig. 3A) and one juvenile Hazel Grouse (*Tetrastes bonasia;* Phasianidae, Galliformes; Fig. 3B). An individual *T. bonasia* was hunted at Ubaranai site no. 1 (Abashiri City, Hokkaido, Japan; N 43.9678°, E 144.0414°) on 7 November 2013, and two *S. orientalis* were shot at Ubaranai site no. 2 (Abashiri City, Hokkaido, Japan; N 43.9261°, E 144.0406°) on 28 October 2016. These birds were shot by a professional hunter for food and stored in a freezer; we then received them from the hunter and carefully extracted the crop and gizzard contents. Crop and gizzard contents of *T. bonasia* were identified from a photograph, while those of *S. orientalis* were identified directly from samples. In addition, the combined weight of crop and gizzard contents were measured for both *S. orientalis* individuals using an electronic scale (wet and dry weights for one, and dry weight only for the other; Online-only Table 2). The data collected from these samples is also included in our database.

## Data Records

The database for the ecological traits of birds in Japan is available on Zenodo^40^ with the original database in XLSX format (*i.e*. “Whole_Database.xlsx” on Zenodo), the reference list correspond to the database in PDF and CSV format (*i.e*. “Reference_List.pdf” and “Reference_List.csv” on Zenodo), and ten different data files for each trait category with described names provided in CSV format. For CSV format files on Zenodo, we used “0” and “1” instead of “−” and “ + ” in original database, respectively, “Unknown” for the missing data of mid three traits (*i.e*. variable no. 4, 5 and 6) like as in original database, and “NULL” for the non-existing values on the last four variables (*i.e*. variable no. 7, 8, 9 and 10). We also included the literary references as numbers in the data files as shown in “Reference_List.pdf” and “Reference_List.csv” on Zenodo. The first row of each CSV data file shows the description of each file, the second row includes a header indicating the names of variables, and the following rows present data for each bird species in Japan. The crop and gizzard contents from two individuals of *S. orientalis* used in this study were stored in Bihoro Museum (Bihoro, Hokkaido, Japan; Specimen IDs: BIHM0300372 and BIHM0300373).

## Technical Validation

All records included in the database are based on articles in scientific journals and books; therefore we have confidence in their accuracy. We also listed the references cited in the database, making it possible for users to access the original sources. Moreover, some bird specialists, MK and T-Squires and mollusc specialists, Y-Morii, T-Saito and DY have carefully checked the database for possible errors and included information on outdated or updated avian binomials in Online-only Table 1.

## Data Availability

No code was used in this study.

## Online-only Tables

**Online-only Table 1:** Information on binomials or species names updated since the 2012 publication of our reference checklist the Check-list of Japanese Birds, 7th Revised Edition.

Species name	Updated binomial(s)	Special notes
*Anas querquedula*	*Spatula querquedula*	—
*Anas formosa*	*Sibirionetta formosa*	—
*Melanitta fusca*	*Melanitta stejnegeri*	—
*Pterodroma phaeopygia*	*Pterodroma sandwichensis*	—
*Puffinus pacificus*	*Ardenna pacifica*	—
*Puffinus bulleri*	*Ardenna bulleri*	—
*Puffinus griseus*	*Ardenna grisea*	—
*Puffinus tenuirostris*	*Ardenna tenuirostris*	—
*Puffinus creatopus*	*Ardenna creatopus*	—
*Puffinus carneipes*	*Ardenna carneipes*	—
*Puffinus lherminieri*	*Puffinus bannermani*	—
*Grus vipio*	*Antigone vipio*	—
*Rallus aquaticus*	*Rallus indicus*	—
*Porzana pusilla*	*Zapornia pusilla*	—
*Porzana fusca*	*Zapornia fusca*	—
*Porzana paykullii*	*Zapornia paykullii*	—
*Porzana cinerea*	*Amaurornis cinerea*	—
*Caprimulgus indicus*	*Caprimulgus jotaka*	—
*Heteroscelus brevipes*	*Tringa brevipes*	—
*Heteroscelus incanus*	*Tringa incana*	—
*Eurynorhynchus pygmeus*	*Calidris pygmaea*	—
*Limicola falcinellus*	*Calidris falcinellus*	—
*Tryngites subruficollis*	*Calidris subruficollis*	—
*Philomachus pugnax*	*Calidris pugnax*	—
*Procelsterna cerulea*	*Anous ceruleus*	—
*Larus genei*	*Chroicocephalus genei*	This binomial change is widely accepted, but not yet recognized by the IUCN
*Larus philadelphia*	*Chroicocephalus philadelphia*	This binomial change is widely accepted, but not yet recognized by the IUCN
*Larus brunnicephalus*	*Chroicocephalus brunnicephalus*	This binomial change is widely accepted, but not yet recognized by the IUCN
*Larus ridibundus*	*Chroicocephalus ridibundus*	This binomial change is widely accepted, but not yet recognized by the IUCN
*Larus saundersi*	*Saundersilarus saundersi*	—
*Larus minutus*	*Hydrocoloeus minutus*	—
*Larus atricilla*	*Leucophaeus atricilla*	This binomial change is widely accepted, but not yet recognized by the IUCN
*Larus pipixcan*	*Leucophaeus pipixcan*	This binomial change is widely accepted, but not yet recognized by the IUCN
*Larus ichthyaetus*	*Ichthyaetus ichthyaetus*	This binomial change is widely accepted, but not yet recognized by the IUCN
*Larus thayeri*	*Larus glaucoides*	This species has been demoted to a subspecies of the new binomial
*Sterna caspia*	*Hydroprogne caspia*	—
*Sterna bergii*	*Thalasseus bergii*	—
*Sterna bengalensis*	*Thalasseus bengalensis*	—
*Sterna albifrons*	*Sternula albifrons*	—
*Sterna aleutica*	*Onychoprion aleuticus*	—
*Sterna lunata*	*Onychoprion lunatus*	—
*Sterna fuscata*	*Onychoprion fuscatus*	—
*Buteo buteo*	*Buteo japonicus*	—
*Aquila clanga*	*Clanga clanga*	—
*Otus lempiji*	*Otus semitorques*	—
*Ninox scutulata*	*Ninox japonica*	—
*Todiramphus miyakoensis*	*Todiramphus cinnamomina*	This species has been demoted to a subspecies of the new binomial
*Dendrocopos kizuki*	*Yungipicus kizuki/Picoides kizuki*	This species’ binomial has been changed with conflicting results: BOW uses the prior IUCN recognizes the latter
*Dendrocopos minor*	*Dryobates minor*	—
*Sapheopipo noguchii*	*Dendrocopos noguchii*	—
*Coracina melaschistos*	*Lalage melaschistos*	—
*Coracina divaricatus*	*Pericrocotus divaricatus/Pericrocotus tegimae*	This species has been split into two species, both present in Japan
*Lanius excubitor*	*Lanius borealis*	—
*Remiz pendulinus*	*Remiz consobrinus*	—
*Poecile varius*	*Sittiparus varius*	—
*Calandrella cheleensis*	*Alaudala cheleensis/Alaudala rufescens*	This species’ binomial has been changed with conflicting results: BOW uses the prior IUCN recognizes the latter
*Riparia paludicola*	*Riparia chinensis*	—
*Hirundo daurica*	*Cecropis daurica*	—
*Cettia diphone*	*Horornis diphone*	—
*Locustella fasciolata*	*Locustella amnicola*	—
*Acrocephalus aedon*	*Arundinax aedon*	—
*Zoothera sibirica*	*Geokichla sibirica*	—
*Cichlopasser terrestris*	*Zoothera terrestris*	—
*Turdus naumanni*	*Turdus eunomus*	This species has been split into the present binomial, although *Turdus naumanni* is also extant in Japan
*Luscinia akahige*	*Larvivora akahige*	—
*Luscinia komadori*	*Larvivora komadori*	—
*Luscinia calliope*	*Calliope calliope*	—
*Luscinia cyane*	*Larvivora cyane*	—
*Luscinia sibilans*	*Larvivora sibilans*	—
*Saxicola torquatus*	*Saxicola maurus*	This binomial change is widely accepted, but not yet recognized by the IUCN
*Passer rutilans*	*Passer cinnamomeus*	—
*Motacilla flava*	*Motacilla tschutschensis*	—
*Carduelis spinus*	*Spinus Spinus*	—
*Carduelis flammea*	*Acanthis flammea*	−
*Carduelis hornemanni*	*Acanthis hornemanni*	The IUCN doesn’t recognize this species as separate from *Acanthis flammea*, although it aknowledges the genus change
*Uragus sibiricus*	*Carpodacus sibiricus*	—
*Chaunoproctus ferreorostris*	*Carpodacus ferreorostris*	—

**Online-only Table 2:** Crop and gizzard contents of two bird species investigated in this study.

Contents	*Streptopelia orientalis* (No.1)			*Streptopelia orientalis* (No.2)		*Tetrastes bonasia*
	No. of individuals	Wet weight (g)	Dry weight (g)	No. of individuals	Dry weight (g)	+/−
**Animal matter (land snails)**						
*Cochlicopa lubrica*	1	−	−	−	−	−
*Discus pauper*	3	−	−	3	−	−
*Karaftohelix* (*Ezohelix*) *gainesi*	−	−	−	2	−	−
*Parakaliella affinis*	1	−	−	−	−	−
*Succinea lauta*	−	−	−	−	−	+
Land snails in total	5	0.10	0.05	5	0.05	
**Plant matter (seeds or fruits)**						
*Persicaria thunbergii* var. *thunbergii*	140	1.11	0.80	421	2.43	−
*Schizopepon bryoniifolius*	12	1.10	0.68	42	2.21	−
*Vitis coignetiae*	−	−	−	−	−	+
*Fraxinus mandshurica*	−	−	−	−	−	+
Unknown	1	0.01	0.01	1	0.04	+
Plant matter in total	153	2.22	1.49	464	4.68	
In total	158	2.32	1.54	469	4.73	
Ratio of land snails in all contents (%)	3.2	4.3	1.7	1.1	1.1	
